# Differential association for *N*-acetyltransferase 2 genotype and phenotype with bladder cancer risk in Chinese population

**DOI:** 10.18632/oncotarget.9475

**Published:** 2016-05-19

**Authors:** Lei Quan, Koushik Chattopadhyay, Heather H. Nelson, Kenneth K. Chan, Yong-Bing Xiang, Wei Zhang, Renwei Wang, Yu-Tang Gao, Jian-Min Yuan

**Affiliations:** ^1^ University of Pittsburgh Cancer Institute, and Department of Epidemiology, Graduate School of Public Health, University of Pittsburgh, Pittsburgh, Pennsylvania, USA; ^2^ Current affiliation: School of Bioscience and Bioengineering, Guangdong Provincial Key Laboratory of Fermentation and Enzyme Engineering, South China University of Technology, Guangzhou China; ^3^ Masonic Cancer Center, and Division of Epidemiology and Community Health, School of Public Health, University of Minnesota, Minneapolis, Minnesota, USA; ^4^ Comprehensive Cancer Center, The Ohio State University, Columbus, Ohio, USA; ^5^ Shanghai Cancer Institute, Renji Hospital, Shanghai Jiaotong University School of Medicine, Shanghai, China

**Keywords:** NAT2, N-acetylation, O-acetylation, bladder cancer, case-control

## Abstract

**Background:**

N-acetyltransferase 2 (NAT2) is involved in both carcinogen detoxification through hepatic *N*-acetylation and carcinogen activation through local *O*-acetylation. NAT2 slow acetylation status is significantly associated with increased bladder cancer risk among European populations, but its association in Asian populations is inconclusive.

**Methods:**

NAT2 acetylation status was determined by both single nucleotide polymorphisms (SNPs) and caffeine metabolic ratio (CMR), in a population-based study of 494 bladder cancer patients and 507 control subjects in Shanghai, China.

**Results:**

The CMR, a functional measure of hepatic *N-*acetylation, was significantly reduced in a dose-dependent manner among both cases and controls possessing the SNP-inferred NAT2 slow acetylation status (all *P-*values<5.0×10^−10^). The CMR-determined slow *N*-acetylation status (CMR<0.34) was significantly associated with a 50% increased risk of bladder cancer (odds ratio = 1.50, 95% confidence interval = 1.10-2.06) whereas the SNP-inferred slow acetylation statuses were significantly associated with an approximately 50% decreased risk of bladder cancer. The genotype-disease association was strengthened after the adjustment for CMR and was primarily observed among never smokers.

**Conclusions:**

The apparent differential associations for phenotypic and genetic measures of acetylation statuses with bladder cancer risk may reflect dual functions of NAT2 in bladder carcinogenesis because the former only measures the capacity of carcinogen detoxification pathway while the latter represents both carcinogen activation and detoxification pathways. Future studies are warranted to ascertain the specific role of *N*- and *O*-acetylation in bladder carcinogenesis, particularly in populations exposed to different types of bladder carcinogens.

## INTRODUCTION

Urinary bladder cancer is the fourth most common cancer in men of the United States [1]. Known risk factors include increasing age, male sex, certain occupational exposures and cigarette smoking. Cigarette smoking is a major cause for bladder cancer, accounting for approximately 50% of all incident cases [2]. Carcinogenic arylamines present in tobacco smoke, including 4-aminobiphenyl (4-ABP) and 2-naphthylamine are believed to be responsible for the increased risk of bladder cancer among smokers [3].

**Figure 1:**
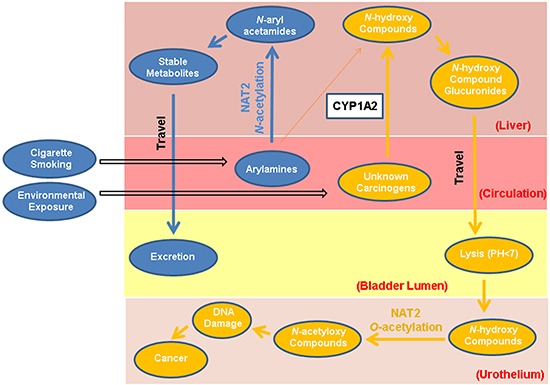
Dual functions of NAT2 in bladder carcinogenesis Potential dual functions of the NAT2 enzyme involve carcinogenic detoxification (*N*-acetylation) and activation (*O*-acetylation). Arylamines from cigarette smoke and other occupational or environmental sources can be *N*-acetylated by hepatic NAT2 function, or possibly ring-oxidized by CYP1A2. The derivatives can form more stable metabolites through conjugation catalyzed by glutathione S-transferases, as well as UDP-glucuronosyltransferases, and ultimately excreted through urine. Alternatively, arylamines and other unknown bladder carcinogens could be procarcinogens that require metabolic activation to electrophilic intermediates to exert their carcinogenic effect. The first step in this process is *N*-oxidation catalyzed by cytochrome P450 (CYP) enzymes, such as CYP1A2 in the liver, to form metabolically active *N*-hydroxy- compounds. The *N*-hydroxy-compounds are transported through circulation to the bladder lumen, where they can be *O*-acetylated by local NAT2 or hydrolyzed in the acidic environment to form highly electrophilic derivatives that can covalently bind to urothelial DNA or generate reactive oxygen species (ROS). Certain potential bladder carcinogens such as heterocyclic amines (HCAs) do not go through the detoxifying *N*-acetylation pathway.

Many environmental and occupational carcinogens undergo catalysis by *N*-acetyltransferase 2 (NAT2). The enzyme detoxifies carcinogens such as arylamines in the liver by transferring an acetyl group to an exocyclic amine (*N*-acetylation). Alternatively, NAT2 may activate carcinogens by transferring an acetyl group to the oxygen group of *N*-hydroxy-aromatic or heterocyclic amines (*O*-acetylation) [4] (Figure 1). Hepatic NAT2 *N*-acetylation capacity can be determined phenotypically by quantifying the urinary caffeine metabolite ratio (CMR) [5]. More recently, the NAT2 acetylation status can be inferred by a panel of seven single nuclear polymorphisms (SNPs) [6] or a tag SNP rs1495741 of the *NAT2* gene [7]. The genetic variants may represent both NAT2 *N*-acetylation status in the liver and *O*-acetylation activity in the urinary tract. Currently, there is no method available for direct measure of local *O*-acetylation activity *in vivo*.

Epidemiological studies in Europe and United States, especially those among smokers, have reported associations between SNP-inferred NAT2 slow acetylation status and increased risk of bladder cancer. It is consistent with the hypothesis that NAT2 plays an important role in detoxifying arylamines present in tobacco smoke through hepatic *N*-acetylation activities [8–12]. However, in two studies of factory workers in China who were occupationally exposed to benzidine, a group I human bladder carcinogen classified by the International Agency for Research on Cancer (IARC) [13], SNP-inferred NAT2 slow acetylation was associated with a significant decreased risk of bladder cancer [14, 15]. It has been hypothesized that NAT2 enzyme may activate rather than deactivate benzidine at the urinary bladder [15], suggesting a more complex function of NAT2. Tobacco smoking is a major identified risk factor for bladder cancer, but it only accounts for 50-60% of disease burden [2]. Thus exposure to diffused environmental bladder carcinogens as yet to be identified may contribute to the remaining 40-50% of disease burden [16]. Furthermore, the incidence rate of bladder cancer in Chinese men is only about one-third that of their Caucasian counterparts, despite much higher prevalence of cigarette smoking in the former than the latter [17, 18]. On the other hand, the proportion of SNP-inferred NAT2 slow acetylators is considerably lower in Chinese (~19%) than in Caucasians (~55%) [9, 19–21]. Such difference certainly contributes to the difference in the bladder cancer rates between the two races. Thus, the inconsistent results of the NAT2 acetylation status and bladder cancer risk from epidemiological studies in different races could be the results of different prevalence of genetic variants in the *NAT2* gene and different levels of exposure to tobacco and other environmental bladder carcinogens.

There are few reports that examined the association between NAT2 acetylation status and bladder cancer risk among Asian populations. Their results were inconclusive [9, 22–27]. Most of these studies were hospital-based and with relatively small sample sizes. None have studied NAT2 acetylation status simultaneously inferred by genotype and phenotype in relation to risk of bladder cancer. The present study was designed to address these limitation and to examine and further clarify the following associations: (1) the concordance of acetylation status inferred by the conventional *NAT2* SNP panel and the novel tag SNP of *NAT2* gene in a Chinese population, (2) the causal relationship between the SNP-inferred acetylation status and CMR, a functional measure of hepatic *N*-acetylation, and (3) the association between SNP- and CMR-determined NAT2 acetylation status separately or in combination and the risk of bladder cancer in a relatively large population-based case-control study in Shanghai, China.

## RESULTS

Participant characteristics are shown in Table 1. Patients with bladder cancer did not differ significantly from controls in the distributions of sex, age at reference date (2 years before diagnosis for cases or 2 years before interview for controls), body mass index (BMI), and level of education. Cases were more likely to smoke cigarettes than controls. Among smokers, patients with bladder cancer had a significantly younger age at starting to smoke and a greater number of years of smoking than controls.

**Table 1:** Distributions of baseline characteristics in bladder cancer patients (cases) and control subjects (controls), The Shanghai Bladder Cancer Study

Characteristics	Cases	Controls	P*^a^*
**Among all subjects**	(n=478)	(n=473)	
**Age at Reference (Years), mean ± SD**	61.0±9.9	62.1±10.0	0.08
**Body Mass Index (Kg/m^2^), mean ± SD**	22.5±3.2	22.2±3.0	0.25
**Gender, %**			
Male	376 (78.7)	370 (78.2)	0.87
Female	102 (21.3)	103 (21.8)	
**Education, %**			
No Formal Education	42 (8.8)	36 (7.6)	0.76
Primary School	115 (24.1)	116 (24.5)	
High School	264 (55.2)	272 (57.5)	
College and Graduate School	57 (11.9)	49 (10.4)	
**Cigarette smoking, %**			
Never	165 (34.5)	208 (44.0)	0.003
Former	73 (15.3)	79 (16.7)	
Current	240 (50.2)	186 (39.3)	
**Among Current and Former Smokers**	(n=313)	(n=265)	
Age starting to smoke (years), mean ± SD	22.8±7.6	24.7±8.2	0.005
Number of cigarettes per day, mean ± SD	15.9±9.6	15.8± 9.1	0.82
Number of years of smoking, mean ± SD	35.4± 13.0	33.3± 13.2	0.05
Number of pack-years of smoking, mean ± SD	29.7± 22.3	27.6± 20.1	0.24

a 2-sided *P*s were derived from Student *t-*tests for continuous variables and Chi-square test for categorical variables.

After excluding a monomorphic SNP (rs1801279) in the study population, we analyzed 6 candidate SNPs and a tag SNP of the *NAT2* gene. Among control subjects, minor allele frequencies (MAF) ranged from 0.03 to 0.45 (Table 2). SNPs 803A>G (rs1208), 481C>T (rs1799929) and 341T>C (rs1801280) were highly correlated with each other (all pairwise *r*^2^ ≥0.94), whereas a moderate correlation was observed between 282 C>T (rs1041983) and 590 G>A (rs1799930) (*r*^2^=0.44). No correlation was observed between other pairs of the candidate SNPs among controls. The tag SNP rs1495741 was only correlated with 282 C>T (rs1041983) (*r*^2^=0.77) (Supplementary Table S1 and Supplementary Figure S1). Allele frequencies and linkage disequilibrium (LD) observed in the present study population were similar to those of other Chinese populations but very different from those of Caucasians published in the HapMap database (Supplementary Figure S2A, S2B, and S2C).

**Table 2:** The geometric means of urinary caffeine metabolite ratio (CMR) by *NAT2* genotypes in bladder cancer patients (cases) and control subjects (controls), The Shanghai Bladder Cancer Study

NAT2 Genotypes		MAF *^a^*	Cases		Controls		% Diff *^c^*	P*^d^*
			N	Geometric Means of CMR (95% CI) *^b^*	N	Geometric Means of CMR (95% CI) *^b^*		
**All Subjects**		N/A	478	0.42 (0.40-0.44)	473	0.46 (0.44-0.48)	−8.7	0.03
**Individual SNP of 6-SNP Panel**								
282 C>T	CC	0.41	188	0.52 (0.48-0.56)	151	0.50 (0.46-0.54)	4.0	0.48
(rs1041983)	CT		221	0.40 (0.40-0.46)	251	0.48 (0.44-0.50)	−12.5	0.006
	TT		69	0.22 (0.20-0.26)	71	0.32 (0.28-0.36)	−31.3	0.005
	*P-*trend			1.2×10^−32^		2.1×10^−8^		
590 G>A	GG	0.24	293	0.46 (0.44-0.50)	271	0.48 (0.44-0.50)	−4.1	0.44
(rs1799930)	GA		170	0.38 (0.34-0.40)	176	0.44 (0.42-0.48)	−13.6	0.0009
	AA		15	0.24 (0.18-0.30)	26	0.28 (0.22-0.34)	−14.3	0.54
	*P-*trend			2.6×10^−10^		4.0×10^−4^		
857 G>A	GG	0.16	336	0.46 (0.44-0.50)	327	0.46 (0.44-0.50)	0	0.83
(rs1799931)	GA		126	0.36 (0.32-0.38)	137	0.42 (0.38-0.46)	−14.3	0.002
	AA		16	0.22 (0.16-0.26)	9	0.32 (0.22-0.44)	−31.3	0.14
	*P-*trend			4.0×10^−16^		0.01		
803 A>G	AA	0.03	450	0.42 (0.40-0.44)	442	0.46 (0.42-0.48)	−8.7	0.03
(rs1208)	AG		26	0.38 (0.30-0.46)	30	0.42 (0.34-0.50)	−9.5	0.44
	GG		2	0.42 (0.22-0.84)	1	0.08 (0.04-0.24)	N/A ***^e^***	N/A
	*P-*trend			0.27		0.04		
481 C>T	CC	0.03	453	0.42 (0.40-0.44)	443	0.46 (0.44-0.48)	−8.7	0.02
(rs1799929)	CT		24	0.38 (0.32-0.48)	29	0.42 (0.34-0.50)	−4.8	0.68
	TT		1	0.50 (0.20-1.32)	1	0.08 (0.04-0.24)	N/A ***^e^***	N/A
	*P-*trend			0.52		0.05		
341 T>C	TT	0.03	452	0.42 (0.40-0.44)	443	0.46 (0.44-0.48)	−8.7	0.02
(rs1801280)	TC		25	0.38 (0.32-0.46)	29	0.42 (0.34-0.50)	−9.5	0.6
	CC		1	0.50 (0.20-1.34)	1	0.08 (0.04-0.24)	N/A ***^e^***	N/A
	*P-*trend			0.44		0.05		
**6-SNP panel inferred acetylation status**								
	Rapid ***^f^***	N/A	170	0.52 (0.50-0.56)	135	0.50 (0.46-0.54)	4.0	0.28
	Int		231	0.44 (0.42-0.46)	260	0.50 (0.46-0.52)	−12.0	0.0006
	Slow		77	0.24 (0.22-0.26)	78	0.30 (0.26-0.32)	−20.0	0.03
	*P-*trend			4.4×10^−34^		5.6×10^−11^		
**Tag SNP**	GG	0.45	163	0.52 (0.50-0.56)	134	0.50 (0.46-0.54)	4.0	0.23
(rs1495741)	GA		240	0.44 (0.42-0.46)	250	0.50 (0.46-0.52)	−12.0	0.0007
	AA		75	0.24 (0.22-0.26)	89	0.32 (0.28-0.34)	−25.0	0.005
	*P-*trend			2.1×10^−33^		4.4×10^−10^		

a MAF: Minor allele frequency among controls.

b Adjusted for age at reference date and sex.

c Percentage of differences: cases' geometric mean minus controls' geometric mean divided by controls' geometric mean, then multiplied by 100.

d 2-sided *Ps* were derived from analysis of covariance comprising the difference in geometric means of caffeine metabolite ratio between cases and controls with adjustment for age and sex.

e N/A, not applicable due to small number of subjects in this category.

f Acetylation status inferred by genotypes of the 6-candidate SNPs, as described in details in the Methods section; Int: intermediate.

*NAT2* genotypes partially reflected hepatic *N*-acetylation function measured by CMR. Among control subjects, minor alleles of all individual SNPs were associated with lower level of CMR compared to major alleles (Table 2, *P*-trends ≤ 0.05). Similar genotype-phenotype associations were found in bladder cancer patients except for the three SNPs with extremely low MAF. NAT2 acetylation status inferred genotypically by the tag SNP and the 6-SNP panel were highly correlated in both controls (*r^2^*=0.87) and cases (*r^2^*=0.96) (*P* < 1.4×10^−30^) (Supplementary Table S2), indicating that the single tag SNP rs1495741 captured almost all genetic variation of the *NAT2* exon 2 region in Chinese people. The SNP-inferred NAT2 slow acetylation status, either based on the tag SNP or 6-SNP panel, resulted in a similar ~40% reduction in CMR in both controls and cases (*P*-trends ≤ 4.4×10^−10^) (Table 2). Smoking, gender and age did not modify the influencing effects of genetic variants on the CMR (Supplementary Table S3).

Patients with bladder cancer overall had statistically significant 8.7% lower CMR than controls (P = 0.03) (Table 2). Compared to their counterparts in control group, the CMR was 12% and ≥20% lower in case patients with intermediate and slow acetylation status inferred by *NAT2* genotype (both *P* values < 0.03). Among subjects with the SNP-inferred NAT2 rapid acetylation status, however, cases had a 4% higher in CMR than controls, but the difference was not statistically significant (*P* = 0.28).

Overall SNP-inferred slow acetylation status was significantly associated with reduced risk of bladder cancer compared with rapid acetylation status (Table 3). This risk association was slightly stronger for acetylation status inferred by the tag SNP (*P*-trend < 0.03) than by the 6-SNP panel approach (*P*-trend = 0.06). On the other hand, low level of CMR (i.e., slow *N*-acetylation status) was associated with an increased risk of bladder cancer compared to high level of CMR (Table 3). Controlling for variation in hepatic *N-*acetylation capacity (i.e., CMR) significantly strengthened the association between SNP-inferred acetylation status and bladder cancer risk; the slow acetylation status was associated with approximately 50% reduced risk of bladder cancer compared with the rapid acetylation status (*P*-trends ≤ 0.003). Conversely, the adjustment for SNP-determined acetylation status also strengthened the CMR-bladder cancer risk association (Table 3). No significant collinearity was observed between *NAT2* genotype and CMR in the same model (tolerance > 0.86). The association between the SNP-inferred acetylation status and bladder cancer risk was much stronger in never smokers than that in ever smokers. Furthermore, the association between SNP-inferred acetylation status and bladder cancer became even stronger in lifelong never smokers who were not exposed to environmental tobacco smoke (ETS), determined by non-detectable total cotinine in urine (data not shown).

**Table 3:** Associations between NAT2 acetylation status inferred by genotype and bladder cancer risk, The Shanghai Bladder Cancer Study

NAT2 Status	All Samples			Never Smokers			Ever Smokers		
	Ca/Co*^a^*	OR (95%CI) *^b^*	OR (95%CI)*^c^*	Ca/Co	OR (95%CI) *^b^*	OR (95%CI)*^c^*	Ca/Co	OR (95%CI) *^b^*	OR (95%CI)*^c^*
**6-SNP Panel**									
Rapid ***^d^***	170/135	1.00	1.00	65/64	1.00	1.00	105/71	1.00	1.00
Intermediate	231/260	0.70(0.52-0.94)	0.69(0.52-0.93)	81/108	0.74(0.47-1.16)	0.72(0.46-1.14)	150/152	0.66(0.45-0.97)	0.66(0.45-0.97)
Slow	77/78	0.75(0.51-1.11)	0.55(0.35-0.85)	19/36	0.51(0.26-0.98)	0.31(0.14-0.67)	58/42	0.93(0.56-1.55)	0.74(0.42-1.31)
*P* for trend		0.06	0.003		0.04	0.004		0.48	0.12
**Tag SNP rs1495741**									
GG (rapid)	163/134	1.00	1.00	64/63	1.00	1.00	99/71	1.00	1.00
GA (Intermediate)	240/250	0.79(0.59-1.06)	0.78(0.58-1.04)	82/107	0.76(0.48-1.19)	0.75(0.47-1.18)	158/143	0.79(0.54-1.17)	0.78(0.53-1.15)
AA (Slow)	75/89	0.66(0.45-0.98)	0.46(0.30-0.72)	19/38	0.48(0.25-0.93)	0.29(0.13-0.62)	56/51	0.80(0.49-1.31)	0.60(0.34-1.04)
*P* for trend		0.03	0.0009		0.03	0.006		0.31	0.06
**Caffeine metabolite ratio**									
Rapid(CMR≥ 0.34)	355/384	1.00	1.00	129/175	1.00	1.00	226/209	1.00	1.00
Slow(CMR< 0.34)	123/89	1.50(1.10-2.06)	1.98(1.37-2.85)	36/33	1.55(0.91-2.62)	2.54(1.34-4.82)	87/56	1.49(1.00-2.20)	1.74(1.11-2.71)
*P*		0.01	0.0003		0.11	0.005		0.05	0.05

Abbreviations: Int, intermediate; OR, odds ratio; CI, confidence interval; CMR, caffeine metabolite ratio.

a Number of bladder cancer patients (Ca) and control subjects (Co).

b Adjusted for age at reference date (continuous) and sex. Additionally adjusted for smoking status (never, former, current), number of cigarettes per day (continuous), and number of years of smoking (continuous) for all subjects and ever smokers.

c In addition to variables in ^b^, hepatic *N-*acetylation status determined by CMR (<0.34 versus ≥0.34) was adjusted for the genotype-disease association; and genotype of Tag SNP rs1495741 was adjusted for the CMR-disease association.

d The acetylation status inferred by the 6-candidate SNP genotypes of *NAT2*, as described in details in the Methods section.

We further examined the associations between the SNP-inferred acetylation status and bladder cancer risk stratified by CMR levels (Table 4). Among subjects with CMR ≥ 0.34, the SNP-inferred slow acetylation status was associated with a statistically significantly reduced risk of bladder cancer; ORs (95% CIs) for the slow acetylation status inferred by the 6-SNP panel and the tag SNP were 0.31 (0.16-0.61) and 0.25 (0.13-0.48), respectively (both *P-*trends ≤ 6.0×10^−5^). The associations were not strengthened by further stratification by smoking status, possibly due to limited number of subjects in each category (data not shown). In contrast, among subjects with CMR < 0.34, the SNP-inferred slow acetylation status was associated with statistically non-significant increase in risk of bladder cancer. The difference in the genotype-disease relationship between the high and low CMR groups was statistically significant (*P*-interactions ≤ 0.01).

**Table 4:** The association between *NAT2* genotype-inferred acetylation status and risk of bladder cancer stratified by hepatic *N*-acetylation status determined by caffeine metabolic ratio (CMR), The Shanghai Bladder Cancer Study

*NAT2* genotype	Rapid hepatic *N-*acetylators (CMR ≥ 0.34)					Slow hepatic *N-*acetylators (CMR < 0.34)				
	Cases		Controls		OR (95%CI)^b^	Cases		Controls		OR (95%CI)*^b^*
	N	CMR^a^	N	CMR^a^		N	CMR*^a^*	N	CMR*^a^*	
**6-SNP Panel**										
Rapid ***^c^***	151	0.58	116	0.56	1.00	19	0.22	19	0.18	1.00
Intermediate	189	0.50	232	0.52	0.62 (0.45-0.85)	42	0.24	28	0.22	1.48 (0.65-3.35)
Slow	15	0.44	36	0.54	0.31 (0.16-0.61)	62	0.20	42	0.16	1.44 (0.66-3.14)
*P* for trend		1.2 × 10^−14^		0.03	6.0 × 10^−5^		0.14		0.21	0.43
	***P*** interaction**=**0.01									
**Tag SNP rs1495741**										
GG (rapid)	145	0.58	116	0.56	1.00	18	0.22	18	0.18	1.00
GA (Intermediate)	196	0.50	224	0.52	0.70 (0.51-0.95)	44	0.24	26	0.22	1.73 (0.75-4.01)
AA (Slow)	14	0.44	44	0.54	0.25 (0.13-0.48)	61	0.20	45	0.16	1.33 (0.60-2.95)
*P* for trend		1.2 × 10^−13^		0.09	4.0 × 10^−5^		0.19		0.36	0.54
	***P*** interaction=0.008									

Abbreviations: Int, intermediate; OR, odds ratio; CI, confidence interval.

a Adjusted for age at reference date (continuous) and sex.

b Adjusted for age at reference date (continuous), sex, smoking status (never, former, current), number of cigarettes per day (continuous), and years of smoking (continuous).

c NAT2 acetylation status inferred by the 6-candidate SNP genotypes of *NAT2*, as described in details in the Methods section.

## DISCUSSION

The present study with a relatively large sample size in an Asian population demonstrates several novel findings. 1) The acetylation status inferred by a single tag SNP captures almost all genetic variations in the exon 2 region of the *NAT2* gene in Chinese people; 2) Both the single tag SNP and multiple candidate SNPs reflect variation in CMR, a measure of hepatic *N-*acetylation capacity; 3) The SNP-inferred slow acetylation status is significantly associated with a reduced risk of bladder cancer whereas the CMR-determined slow acetylation status is significantly associated with increased risk of bladder cancer as reported previously in this study population [20] as well as in Western populations [19]. The association between the SNP-inferred slow acetylation status and reduced risk of bladder cancer becomes stronger when the inter-individual variation in CMR is controlled for, suggesting that the *O-*acetylation rather than *N-*acetylation plays an important role in the observed SNP-bladder cancer risk association. Conversely, the effect of CMR-determined slow acetylation status on bladder cancer risk was greatly increased after controlling for genetic variation in the *NAT2*, further suggesting hepatic *N-*acetylation measured by CMR is a detoxification pathway of bladder carcinogen arylamines. 4) The SNP-inferred slow acetylation and reduced risk of bladder cancer was mainly confined to never smokers, further suggesting the potential role of NAT2 in the activation of non-tobacco bladder carcinogens. These novel and intriguing findings demonstrate a more complex function of the *NAT2* gene in bladder carcinogenesis than previously appreciated.

Urinary CMR primarily measures hepatic *N*-acetylation activity of NAT2 whereas *NAT2* genotypes potentially reflect both *N*- and *O*-acetylation activities [6, 28, 29]. Dual functions of NAT enzymes that involve carcinogenic detoxification (*N*-acetylation) and activation (*O*-acetylation) have been long described in the liver [30, 31]. It has been shown in various animal models and in human tissue that functional levels of NAT2 expression are also detected in the urinary bladder [32–34]. The literature is mixed on NAT2 catalytic activity in the urinary bladder, which is partially due to the strong NAT1 catalytic activity in the tissue [34]. However, NAT2-dependent 4-ABP and 2-naphthylamine *N*-acetyltransferase activities have been reported in human bladder [28, 35, 36]. Moreover, p-Aminobenzoic acid *N*-acetyltransferase activity (selective for NAT1) and *N*-hydroxy-ABP *O*-acetyltransferase activities (not selective for NAT1 or NAT2) in human bladder cytosols do not correlate [35]. These findings provide compelling evidence for the existence of NAT2 *O*-acetylation function in the local urothelium.

The increased risk of bladder cancer associated with low CMR observed in the present study as well as by others supported an important role of hepatic *N-*acetylation in the detoxification of bladder carcinogens [9, 19, 20, 37]. On the other hand, most *NAT2* variant alleles share one or more common missense SNPs that decrease gene expression, enzyme activity or enzyme stability that affect both *N*- or *O*-acetylation functions [5, 38]. Therefore, *NAT2* SNP-inferred slow acetylation status potentially measured variations in both *N*- and *O*-acetylation catalytic capacities [28], and was associated with decreased risk of bladder cancer in our study. The adjustment for CMR resulted in significant strengthening of the association, which indirectly demonstrated an important role of *O-*acetylation in the activation of bladder carcinogens, leading to elevated risk of bladder cancer for individuals who carried high functional alleles of the *NAT2* gene. This was further supported by a stronger association between the SNP-inferred slow acetylation status and decreased bladder cancer risk among individuals with higher CMR.

Different carcinogens are targeted by the *N-* and *O-*acetylation pathways [39]. Therefore, the net effect of SNP-inferred NAT2 acetylation status on risk of bladder cancer would depend on the individual's exposure to specific type of bladder carcinogens. Smokers are exposed primarily to arylamines, substrates of the *N-*acetylation pathway. Epidemiological studies and genome-wide association (GWA) studies in Europe consistently reported higher risk of bladder cancer associated with the SNP-inferred NAT2 slow acetylation status among ever smokers [9–12, 23, 40, 41]. Compared to ever smokers, never smokers experience two- to three-fold lower risk of bladder cancer [2], and thus may be more sensitive to diffused environmental carcinogens as substrates of the *O-*acetylation pathway.

Metabolism of heterocyclic amines (HCAs) involves primarily activation through local *O*-acetylation of NAT2 but not detoxification through hepatic *N*-acetylation due to the steric hindrance of the exocyclic amine [5]. Although the findings were not all consistent, NAT2-catalyzed *O*-acetylation of *N*-hydroxy-heterocyclic amines accounted for the association between NAT2 rapid acetylation status and increased risk of colorectal cancer among people who frequently consumed well-done meat [42–45]. A positive association between meat intake and risk of bladder cancer has also been demonstrated among never smokers with genotype-inferred NAT2 rapid acetylation status, suggesting a possible activation effect of NAT2 on HCA through *O-*acetylation pathway in the urinary bladder [46]. Indeed, the fry-cooking of meat, a common food preparation by Chinese, produces high levels of HCAs (~49.95 ng/day) [47]. Future studies are warranted to investigate the interaction between NAT2 and exposure to HCA carcinogens on risk of bladder cancer.

Notable strengths of the study included population based design, relatively large sample size and homogenous study population (only Han Chinese). We collected detailed information on various aspects of participants' characteristics and controlled potential confounding effects such as smoking. Furthermore, urinary total cotinine was quantified and active and passive smoking status was confirmed. More importantly, the function of the SNPs in the *NAT2* gene was confirmed by CMR, a functional measure of hepatic *N*-acetylation. The simultaneous measurements of both CMR and the *NAT2* SNPs (reflecting both *N-* and *O-*acetylation) allowed for the assessment of the putative association between the *O-*acetylation status and bladder cancer, which provides insight on the potential biological mechanism of *NAT2* genetic variants on bladder carcinogenesis.

Our study has several potential limitations. One of limitations was the retrospective study design. Urine samples were collected from patients after their diagnosis of bladder cancer. The disease status or progress might have potential impact on hepatic function of caffeine metabolism. However, our data did not show any significant difference in CMR by disease stage. Among bladder cancer patients, the CMR levels were comparable across different patients by tumor stages and grades at diagnosis (Supplementary Table S3). Another concern about the retrospective study design was the potential impact of lifestyle changes due to bladder cancer diagnosis on the hepatic *N-*acetylation function. There was a high proportion of patients with bladder cancer quit smoking after their cancer diagnosis (Supplementary Table S3). However, smoking status for cases was determined at two years prior to cancer diagnosis. Furthermore, we did not detect any difference in CMR between smoking statuses at urine collection among either controls or cases (Supplementary Table S3). Genotype of the *NAT2* gene would not be altered by the disease status, thus the observed association between the SNP-inferred acetylation status and bladder cancer risk are unlikely to be confounded by the retrospective study design. Nonetheless, the observed association may be due to genetic linkage to other as-yet-unidentified risk genes for bladder cancer.

In summary, this study demonstrates a statistically significant reduced risk of bladder cancer associated with the SNP-inferred NAT2 slow acetylation status, but an increased risk of bladder cancer with the CMR-determined slow acetylation status. The apparent opposite direction for phenotypic and genetic measures of acetylation statuses with bladder cancer risk may reflect dual functions of NAT2 in bladder carcinogenesis because the CMR only measures the systemic detoxification capacity through the hepatic *N*-acetylation pathway while the SNP-inferred NAT2 acetylation represents both hepatic detoxification and local carcinogen activation through the *O-*acetylation pathway at local urothelium. The observed net protective effect of the SNP-inferred NAT2 slow acetylation status on bladder cancer risk may reflect both the high prevalence of *NAT2* rapid acetylation status and exposure to bladder carcinogens that are uniquely catalyzed through the *O-*acetylation activation pathway in the study population. Future studies are warranted to ascertain the specific role of *N*- and *O*-acetylation in bladder carcinogenesis, particularly in populations exposed to different types of bladder carcinogens.

## MATERIALS AND METHODS

### Study participants

The Shanghai Bladder Cancer Study has been described in detail elsewhere [20]. Briefly, bladder cancer patients were identified through the Shanghai Cancer Registry, a population-based cancer registry monitoring approximately 8 million residents in the urban area of Shanghai, China. All cases diagnosed with bladder cancer between July 1^st^ of 1995 and June 30^th^ of 1998 were eligible to participate in the study. The registry identified 708 cases aged 25 to 74 years at diagnosis of bladder cancer, among whom 56 died before contacting, 29 refused to be interviewed and 42 were untraceable. We interviewed the remaining 581 (82%) eligible patients between July 1996 and June 1999. The diagnosis of bladder cancer of 531 (91%) patients was based on histopathological evidence and diagnosis of the remaining 50 (9%) patients was based on positive computerized tomography scan and/or ultrasonography with consistent clinical history.

Control subjects were randomly selected from urban residents of Shanghai through the City Residents Registry. Controls were chosen to match the frequency distribution by sex and 5-year age groups of patients with bladder cancer. Among the 750 randomly selected control subjects, 74 were untraceable and 72 refused to participate in the study. The remaining 604 (80%) subjects were interviewed between July 1996 and June 1999. The study has been approved by institutional review boards at the Shanghai Cancer Institute, the University of Minnesota and the University of Pittsburgh. Consent forms have been signed by all study participants.

### Data collection

In-depth in-person interviews using a structured questionnaire were conducted to collect information from each eligible subject on a variety of factors known or suspected to be related to bladder cancer. The questionnaire included background information, demographics, history of tobacco smoke, history of passive smoking (for non-smokers only), history of alcohol, coffee, tea, soft drinks and plain water drinking, use of hormone replacement therapy (for women only), medical history, usual adult diet and occupational history, from birth to up to 2 years before the diagnosis of bladder cancer for cases and 2 years before the date of interview for controls [20].

All study participants were asked to donate blood and urine samples at the end of the interview. One 10-ml blood sample was collected from each study participant in a heparin coated tube and fractioned into plasma, buffy coat and erythrocytes on the day of the sample collection. All components of the blood samples were stored at −80°C. For the collection of overnight urine sample, each subject was given two packets of Nestle instant coffee or two cans of Coca-Cola classic drink (about 70 mg of caffeine) to be drunk between 3 pm and 6 pm. An overnight urine sample was collected by the subject, picked up by the interviewer in the following morning, processed, acidified (400 mg of ascorbic acid per 20 ml of urine) and stored at −80°C until analysis. A total of 535 (92%) of the 581 interviewed cases and 543 (90%) of the 604 interviewed control subjects donated blood and urine samples. After excluding subjects with missing information on any of the SNPs genotyped (3 cases and 2 controls) or urinary CMR (13 cases and 32 controls), 478 bladder cancer cases and 473 control subjects were included in the present study.

### Laboratory measurements

#### NAT2 phenotype by CMR

The measurement of urinary caffeine metabolites, i.e. 5-acetylamino-6-amino-3-methyluracil (AAMU), 1-methylxanthin (MX), 1-methyluric acid (MU) and 1,7-dimethylxanthin (17X) has been described in detail elsewhere [20]. Briefly, levels of AAMU in urine were determined using high-performance size exclusion chromatography [48]. Quantification of MX, MU and 17X in urine was conducted using a modified method of Grant et al [29]. All analyses were performed with appropriate internal standards. Concentrations of all metabolites were calculated using calibration curves. NAT2 hepatic *N*-acetylation function was determined phenotypically by ratio of urinary caffeine metabolites (CMR) using the following formula: AAMU/(AAMU+ MX+MU). The ratios enabled differentiation at a cut-off of 0.34 and study participants were classified as slow (ratio < 0.34) or rapid (ratio ≥ 0.34) acetylators [48].

Urinary total cotinine was measured by the standard gas chromatographic-mass spectrometric method as described previously [20]. For the subgroup analysis by smoking status at the time of urine collection, we classified study subjects into current smokers and lifelong never smokers. Current smokers were those who self-reported as daily smokers at reference date with urinary total cotinine greater than 75 ng/ml. Lifelong never smokers were self-reported never smokers at reference date with urinary total cotinine <75 ng/ml. Fourteen self-reported never smokers but whose urinary total cotinine were greater than 75 ng/mL were excluded from this subgroup analysis (7 cases and 7 controls). We further classified lifelong never smokers into those were ETS-exposed (urinary total cotinine 1-75 ng/ml) or ETS non-exposed.

#### NAT2 Genotype

Genomic DNA was extracted in batches from peripheral blood buffy coats using QIAmp DNA mini kit according to manufacturer's protocol (Qiagen Inc, Valencia, CA, US). Quality and quantity of purified DNA were evaluated using Nanodrop UV-spectrometer (Thermo Fisher Scientific Inc., Wilmington, DE, US). DNA samples were stored at −80°C until analysis and plated and genotyped at the Genomics Core Facility at University of Minnesota using MassARRAY technology and iPLEX Gold Assay (Sequenom Inc., San Diego, CA, US). Five percent duplicates and two sets of in-house trio samples were included for quality control purposes. The concordance among blind duplicate pairs was >99.95%. The average successful genotyping rate for each sample and SNP was 100%.

A conventional panel of seven SNPs in exon 2 of the *NAT2* gene was used to determine *NAT2* haplotypes and infer acetylation status [49]. The panel includes four SNPs that are known to reduce NAT2 activity: 191G>A (rs1801279), 341T>C (rs1801280), 590 G>A (rs1799930) and 857 G>A (rs1799931); and three SNPs that do not change NAT2 activity but are required to infer acetylation status accurately: 803 A>G (rs1208), 481 C>T (rs1799929) and 282 C>T (rs1041983). One of the seven SNPs, *NAT2* 191G>A (rs1801279), was not polymorphic in our study population and hence was excluded from further analysis. The genotype frequencies for the other six SNPs were all in Hardy-Weinberg equilibrium among the control population. Individuals carrying two of the *NAT2* alleles associated with rapid acetylation status, i.e. NAT2*4 (wild type), NAT2*12A (803 A>G) or NAT2*13A (282 C>T), were classified as rapid acetylators. Individuals carrying two of the *NAT2* alleles associated with slow acetylation status, i.e. NAT2*5A (341T>C and 481 C>T), NAT2*5B (341T>C, 481 C>T and 803 A>G), NAT2*5C (341T>C and 803 A>G), NAT2*6A (282 C>T and 590 G>A), NAT2*7B (282 C>T and 857 G>A) were classified as slow acetylators. Individuals carrying one rapid acetylation allele and one slow acetylation allele were classified as intermediate acetylators [8] (Supplementary Table S4).

In addition to the 6-SNP panel, a tag SNP (rs1495741) recently identified among European populations at the 3′ end of the *NAT2* gene was also genotyped [10]. This SNP has been demonstrated to correlate with NAT2 acetylation status in European populations [7]. The distribution of this SNP was found to be in Hardy-Weinberg equilibrium among control subjects.

### Statistical analysis

Chi-square test was used to examine the difference in distributions of categorical or nominal variables and Student *t-*test for continuous variables between cases and controls. Pearson's correlation coefficient, *r^2^* and corresponding *P* values were calculated for measuring pairwise correlation between two SNPs. Linkage disequilibrium map for the tag SNP rs1495741 in relation to 6 candidate SNPs were generated using Haploview [50]. Analysis of covariance (ANCOVA) method was used to compare the difference in geometric means of CMR between cases and controls, or across different genotypes with adjustment for age and sex.

Unconditional logistic regression was used to calculate odds ratios (ORs), their corresponding 95% confidence intervals (CIs) and associated *P* values to measure the strength of associations between the SNP-inferred or CMR-determined acetylation status and bladder cancer risk with adjustment for age, sex, smoking status, number of cigarettes per day, and number of years of smoking [51]. Similar multivariable logistic regression models with further adjustment for CMR or stratified by CMR were used to assess indirectly the effect of *O*-acetylation on bladder cancer risk. We assumed additive genetic effects and homozygous wild-type, heterozygous, and homozygous variant genotypes were coded as 0, 1, and 2, respectively, in the trend test. To test collinearility between *NAT2* genotype and CMR, the tolerance of each variable in the model was requested, with a value less than 0.20 indicating the presence of collinearity.

All analyses were conducted using SAS 9.4 (SAS Institute, Cary CA). All *P* values were 2-sided. Values of *P* less than 0.05 were considered statistically significant.

## SUPPLEMENTARY FIGURES AND TABLES




