# Supplementary material for: Differential association for N-acetyltransferase 2 genotype and phenotype with bladder cancer risk in Chinese population
Source: Oncotarget. 2016 May 19;7(26):40012–24. doi: 10.18632/oncotarget.9475 (PMC5129988; doi:10.18632/oncotarget.9475)
Supplement: Supplementary file 1 [file oncotarget-07-40012-s001.pdf]

Differential association for *N*-acetyltransferase 2 genotype and phenotype with bladder cancer risk in Chinese population

SUPPLEMENTARY FIGURES AND TABLES

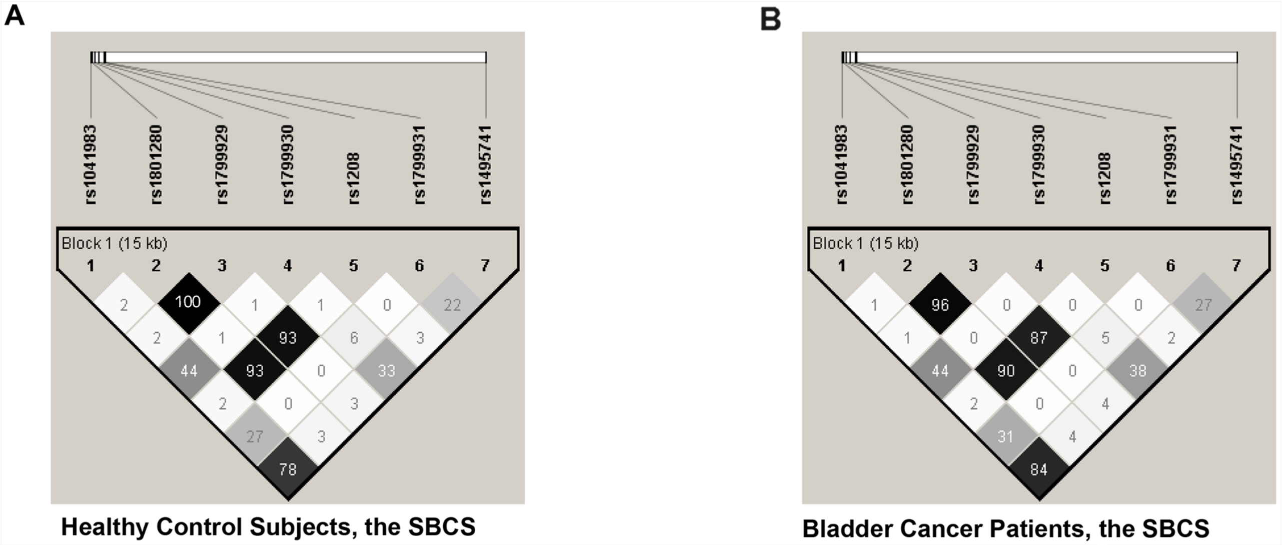

Supplementary Figure S1: Linkage disequilibrium (LD) of conventional 6-panele SNPs and tag SNP rs1495741 in the NAT2 region among cases and controls. The Shanghai Bladder Cancer Study (SBCS). LD structures among cases and controls in the SBCS are shown. Pairwise r2 (x 100) between SNPs is presented in each square. Extent of correlation is indicated by color of the square.

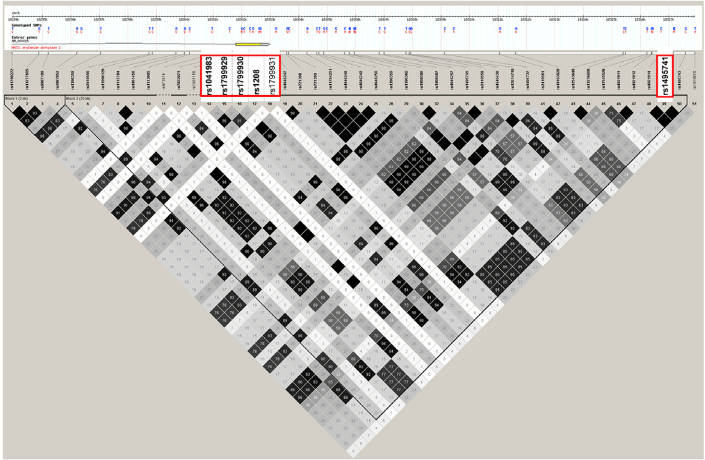

A European Populations in Hapmap Data

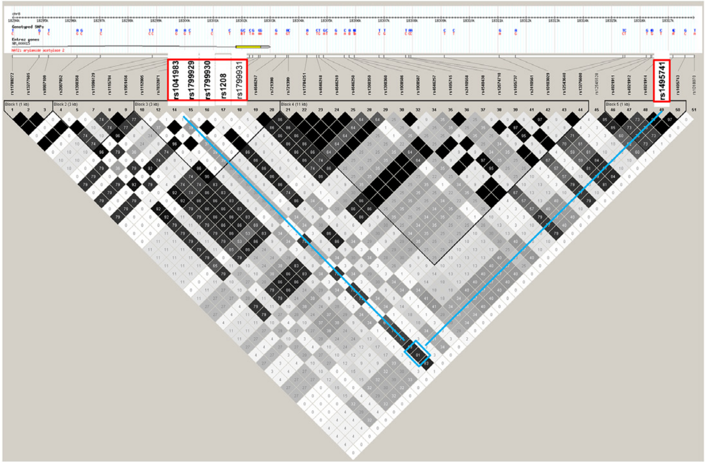

B Chinese Han Population in Hapmap Data

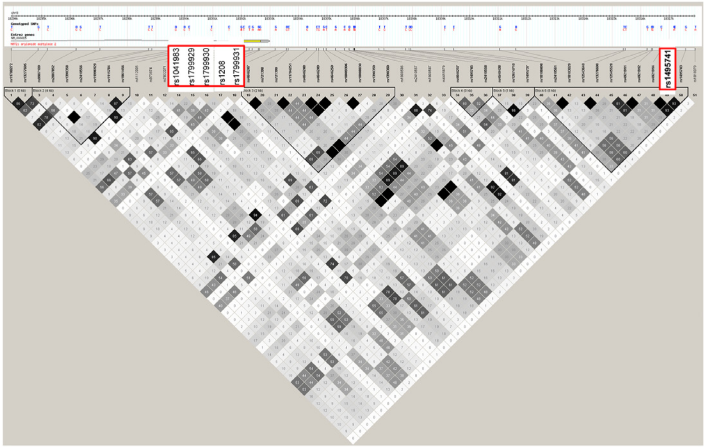

C African Populations in Hapmap Data

Supplementary Figure S2: Linkage disequilibrium (LD) of SNPs in the NAT2 region among different populations in the HapMap database. LD structures in the NAT2 region in European populations **A.** the Chinese Han population **B.** and African populations **C.** based on data of 51 SNPs from the Hapmap database were shown. SNP that were genotyped in SBCS were highlighted in red boxes. Data of rs1801280 was missing in the HapMap database. LD structures in the NAT2 region differed largely across different races. The tag SNP rs1495741 appears to tag the NAT2 region through different mechanism in European populations and in the Chinese population.

Supplementary Table S1: Pairwise r^2 for *NAT2* SNPs genotyped among control subjects, The Shanghai Bladder Cancer Study

	282 C>T (rs1041983)	341 T>C (rs1801280)	481 C>T (rs1799929)	590 G>A (rs1799930)	803 A>G (rs1208)	857 G>A (rs1799931)	Tag SNP (rs1495741)
**282 C>T (rs1041983)**	1.00						
**341 T>C (rs1801280)**	0.02	1.00					
**481 C>T (rs1799929)**	0.02	1.00	1.00				
**590 G>A (rs1799930)**	0.44	0.00	0.00	1.00			
**803 A>G (rs1208)**	0.03	0.94	0.94	0.01	1.00		
**857 G>A (rs1799931)**	0.22	0.01	0.01	0.10	0.01	1.00	
**Tag SNP (rs1495741)**	0.77	0.05	0.05	0.01	0.04	0.18	1.00

Cells corresponding to highly correlated pairs (r^2>0.20) are shaded to facilitate reading.

Supplementary Table S2: The concordance between the acetylation status inferred by the 6-candidate SNPs and the tag SNP rs1495741 of *NAT2*, The shanghai bladder cancer study

**Tag SNP rs1495741**	**Acetylation Status Inferred by 6-Candidate SNPs**		
	**Rapid**	**Intermediate**	**Slow**
Bladder Cancer Cases			
**GG**	163 (95.9%)	0 (0.0%)	0 (0.0%)
**GA**	7 (4.1%)	231 (100.0%)	2 (2.6%)
**AA**	0 (0.0%)	0 (0.0%)	75 (97.4%)
Control Subjects			
**GG**	128 (94.8%)	6 (2.3%)	0 (0.0%)
**GA**	7 (5.2%)	240 (92.3%)	3 (3.9%)
**AA**	0 (0.0%)	14 (5.4%)	75 (96.1%)

Numbers of subjects (column percentage) in each category are shown.

Supplementary Table S3: The distribution of the geometric means of urinary caffeine metabolite ratio (CMR), the Shanghai Bladder Cancer Study.

See Supplementary File 1

Supplementary Table S4: NAT2 acetylation status and *NAT2* haplotypes inferred by genotypes of 6-candiate SNPs

NAT2 Acetylation Status	*NAT2* Haplotypes [a]	282 C>T (rs1041983)	341 T>C (rs1801280)	481 C>T (rs1799929)	590 G>A (rs1799930)	803 A>G (rs1208)	857 G>A (rs1799931)	Cases	Controls
**Rapid (wild type)**	***4A/*4A**	CC	TT	CC	GG	AA	GG	167 (35.0%)	135 (28.5%)
	***4A/*12A**	CC	TT	CC	GG	AG	GG	2 (0.4%)	0 (0.0%)
	***4A/*13A**	CC	TC	CC	GG	AA	GG	1 (0.2%)	2 (0.4%)
**Slow (mutant)**	***5B/*5B**	CC	CC	TT	GG	GG	GG	1 (0.2%)	1 (0.2%)
	***5B/*6A**	CT	CT	TC	GA	GA	GG	3 (0.6%)	10 (2.1%)
	***5B/*7B**	CT	CT	TC	GG	GA	GA	4 (0.8%)	4 (0.9%)
	***5C/*6A**	CT	CT	CC	GA	GA	GG	1 (0.2%)	0 (0.0%)
	***6A/*6A**	TT	TT	CC	AA	AA	GG	15 (3.1%)	26 (5.5%)
	***6A/*7B**	TT	TT	CC	AG	AA	GA	37 (7.8%)	28 (5.9%)
	***7B/*7B**	TT	TT	CC	GG	AA	AA	16 (3.4%)	9 (1.9%)
**Intermediate**	***4A/*5B or *5A/*12A** [b]	CC	TC	CT	GG	AG	GG	16 (3.4%)	15 (3.2%)
**(wt/mut)**	***4A/*6A or *6B/*13A**	CT	TT	CC	GA	AA	GG	129 (27.0%)	132 (27.9%)
	***4A/*7B or *7A/*13A**	CT	TT	CC	GG	AA	GA	83 (17.4%)	102 (21.6%)
	**Other** [c]							3 (0.6%)	9 (1.9%)
	**Total**							478	473

a. *NAT2* haplotypes detected in the study population were shown. *NAT2*5A*, *NAT2*6B* and *NAT2*7A* were not detected.
b. The frequencies of *5A/*12A, *6B/*13A and *7A/*13A are expected to be very low in the study population based on frequencies of their homozygous.
c. Other subjects that are heterozygous for wild-type and mutant alleles.
