# Supplementary material for: Differential association for N-acetyltransferase 2 genotype and phenotype with bladder cancer risk in Chinese population
Source: Oncotarget. 2016 May 19;7(26):40012–24. doi: 10.18632/oncotarget.9475 (PMC5129988; doi:10.18632/oncotarget.9475)
Supplement: Supplementary file 2 [file oncotarget-07-40012-s002.docx]

**Supplementary Table 3. The distribution of the geometric means of urinary caffeine metabolite ratio (CMR), the Shanghai Bladder Cancer Study.**

|  | | **Bladder Cancer Cases** | |  | **Control Subjects** | |
| --- | --- | --- | --- | --- | --- | --- |
|  |  | **No. of Subjects** | **Geometric Means of CMR (95%CI)*^a^*** |  | **No. of Subjects** | **Geometric Means of CMR (95%CI)*^a^*** |
|  | |  |  |  |  |  |
| **All Subjects** | | 478 | 0.42 (0.40-0.44) |  | 473 | 0.46 (0.44-0.48) |
| **General clinical stage** | | | |  |  |  |
|  | I | 208 | 0.44 (0.40-0.46) |  | N/A |  |
|  | II | 114 | 0.40 (0.36-0.44) |  |  |  |
|  | III | 28 | 0.42 (0.32-0.48) |  |  |  |
|  | IV | 7 | 0.50 (0.36-0.74) |  |  |  |
|  | *P-*trend |  | 0.46 |  |  |  |
| **Tumor grade** | |  |  |  | N/A |  |
| Low grade superficial | | 51 | 0.46(0.40-0.54) |  |  |  |
| High grad superficial | | 183 | 0.42 (0.38-0.44) |  |  |  |
| Invasive | | 100 | 0.42 (0.38-0.46) |  |  |  |
| *P-*trend | |  | 0.32 |  |  |  |
| **Time between diagnosis and urine collection** | | | |  | N/A |  |
|  | >0.5 year | 207 | 0.42 (0.38-0.44) |  |  |  |
|  | ≤0.5 year | 274 | 0.42 (0.40-0.46) |  |  |  |
|  | *P* |  | 0.58 |  |  |  |
| **Age at reference** | |  |  |  |  |  |
|  | >64 year | 225 | 0.40 (0.36-0.44)***^b^*** |  | 255 | 0.46 (0.44-0.50) |
|  | ≤64 year | 253 | 0.44 (0.40-0.48) |  | 218 | 0.42 (0.38-0.48) |
|  | *P* |  | 0.13 |  |  | 0.20 |
| **BMI** |  |  |  |  |  |  |
|  | >22 | 248 | 0.42 (0.40-0.46) |  | 253 | 0.44 (0.42-0.48) |
|  | ≤22 | 230 | 0.42 (0.40-0.46) |  | 220 | 0.46 (0.42-0.50) |
|  | *P* |  | 0.92 |  |  | 0.72 |
| **Sex** |  |  |  |  |  |  |
|  | Male | 376 | 0.42 (0.40-0.44)***^c^*** |  | 370 | 0.44 (0.42-0.46) |
|  | Female | 102 | 0.44 (0.40-0.48) |  | 103 | 0.46 (0.42-0.50) |
|  | *P* |  | 0.52 |  |  | 0.58 |
| **Smoking Status** | |  |  |  |  |  |
|  | Never | 165 | 0.44 (0.42-0.48) |  | 208 | 0.46 (0.42-0.48) |
|  | Former | 73 | 0.38 (0.34-0.44) |  | 79 | 0.44 (0.38-0.50) |
|  | Current | 240 | 0.40 (0.38-0.44) |  | 186 | 0.46 (0.42-0.50) |
|  | *P-*trend |  | 0.20 |  |  | 0.88 |
| **Smoking status after cancer diagnosis** ^d^ | | |  |  |  |  |
| Continued to smoke | | 118 | 0.42 (0.38-0.46) |  | N/A |  |
| Quit smoking | | 122 | 0.40 (0.34-0.44) |  |  |  |
| Lifelong nonsmoker | | 158 | 0.46 (0.42-0.48) |  |  |  |
| *P-*trend | |  | 0.20 |  |  |  |

a Adjusted for age at reference date and sex.

b Adjusted only for sex.

c Adjusted only for age at reference date.

d Continued to smoke were those who reported smoking at both reference date (exact 2 years before cancer diagnosis) and at the date of urine collection (median 0.5 year after cancer diagnosis); quit smoking were cases who reported smoking at the reference date but already quit smoking at the date of urine collection; lifelong nonsmoker were those who reported never smoked cigarettes at the reference date and at the date of urine collection and urinary cotinine levels were <75ng/ml. Seventy-three cases who were former smokers and 7 cases who were never smokers at reference date but had urinary total cotinine >75 ng/ml were excluded from the present analysis.
